# Bioinformatics Tools and Databases to Assess the Pathogenicity of Mitochondrial DNA Variants in the Field of Next Generation Sequencing

**DOI:** 10.3389/fgene.2018.00632

**Published:** 2018-12-11

**Authors:** Céline Bris, David Goudenege, Valérie Desquiret-Dumas, Majida Charif, Estelle Colin, Dominique Bonneau, Patrizia Amati-Bonneau, Guy Lenaers, Pascal Reynier, Vincent Procaccio

**Affiliations:** ^1^UMR CNRS 6015-INSERM U1083, MitoVasc Institute, Angers University, Angers, France; ^2^Biochemistry and Genetics Department, Angers Hospital, Angers, France

**Keywords:** mitochondria, mitochondrial diseases, mitochondrial DNA, next generation sequencing, bioinformatics, mtDNA variant interpretation

## Abstract

The development of next generation sequencing (NGS) has greatly enhanced the diagnosis of mitochondrial disorders, with a systematic analysis of the whole mitochondrial DNA (mtDNA) sequence and better detection sensitivity. However, the exponential growth of sequencing data renders complex the interpretation of the identified variants, thereby posing new challenges for the molecular diagnosis of mitochondrial diseases. Indeed, mtDNA sequencing by NGS requires specific bioinformatics tools and the adaptation of those developed for nuclear DNA, for the detection and quantification of mtDNA variants from sequence alignment to the calling steps, in order to manage the specific features of the mitochondrial genome including heteroplasmy, i.e., coexistence of mutant and wildtype mtDNA copies. The prioritization of mtDNA variants remains difficult, relying on a limited number of specific resources: population and clinical databases, and *in silico* tools providing a prediction of the variant pathogenicity. An evaluation of the most prominent bioinformatics tools showed that their ability to predict the pathogenicity was highly variable indicating that special efforts should be directed at developing new bioinformatics tools dedicated to the mitochondrial genome. In addition, massive parallel sequencing raised several issues related to the interpretation of very low mtDNA mutational loads, discovery of variants of unknown significance, and mutations unrelated to patient phenotype or the co-occurrence of mtDNA variants. This review provides an overview of the current strategies and bioinformatics tools for accurate annotation, prioritization and reporting of mtDNA variations from NGS data, in order to carry out accurate genetic counseling in individuals with primary mitochondrial diseases.

## Key Points for *in silico* Prioritization and Interpretation of mtDNA Variants

- Query dedicated mtDNA databases which are regularly updated (e.g., Mitomap, HmtDB).
- Consider the variant's frequency both within the general population and specific haplogroup.
- Detection and interpretation of low heteroplasmy levels should be carefully evaluated.
- Integrate additional information that might modulate the clinical penetrance (e.g., mitochondrial haplogroup, synergistic or helper mtDNA variants, nuclear variants).
- Evaluate inter-species and primates amino-acid or nucleotide conservation.
- Favor *in silico* prediction tools dedicated to mtDNA (e.g., APOGEE, Mitotip, Mtool Box).

## Introduction

**Figure 1:**
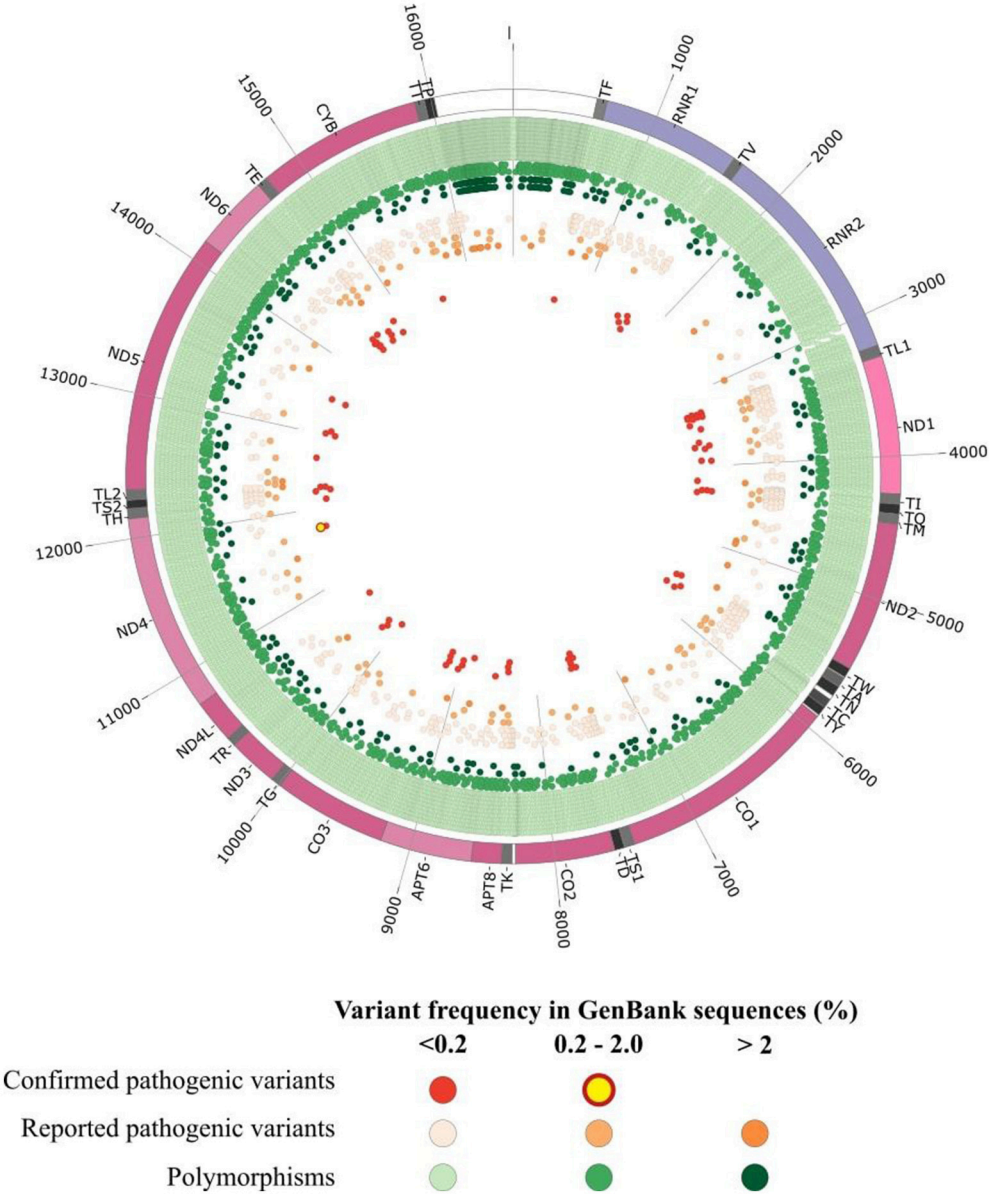
Graphical representation of human mitochondrial DNA variations. The outer circle depicts the mitochondrial genome with annotated tRNAs (gray), rRNAs (purple), protein-coding genes (Bentley et al., 2008), and non-coding regions (white). In the inner circles each point represent an mtDNA variant reported in GenBank sequences collected from Mitomap according to the variant status reported in Mitomap (polymorphisms in green, reported pathogenic variants in orange, confirmed pathogenic variants in red) and variant frequency in GenBank (< 0.2%, light color; 0.2–2.0%, medium color; >2.0%, dark color).

The prevalence of mitochondrial diseases primarily affecting oxidative phosphorylation (OXPHOS) is estimated at about 1 in 4,300 (Gorman et al., 2015). As mitochondrial proteins are encoded both by the nuclear genome and their own genome (mtDNA), their clinical presentation are highly heterogeneous. Human mtDNA is a 16,569 bp circular double-stranded molecule, encoding for 13 polypeptides involved in the oxidative phosphorylation (OXPHOS), together with 2 ribosomal RNAs and 22 tRNAs supporting the translational machinery (Wallace et al., 2010). Pathogenic variants of the mitochondrial genome can affect either the protein coding genes (Wallace et al., 2013), tRNAs (Tang et al., 2013; Gorman et al., 2015) and rRNA genes (Smith et al., 2014; Elson et al., 2015). Genetic defects in the mitochondrial genome can be identified in a variable proportion of patients with mitochondrial respiratory disorders, reaching up to 20% of patients (Thorburn et al., 2004). Hundreds of pathogenic mtDNA variants implicated in a variety of human diseases (Lott et al., 2013) have now been reported in the continuously updated Human Mitochondrial Genome Database—the Mitomap (Ruiz-Pesini et al., 2007; Lott et al., 2013) but as of today (July 2018) only 84 variants have a confirmed status, whereas a total of 595 other variants classified as reported, awaiting a final confirmation of pathogenicity (Figure 1). These mtDNA variants lead to a broad spectrum of maternally-inherited diseases, ranging from lethal neonatal syndromes to multisystemic disorders, with high variable clinical phenotypes and penetrance, mainly resulting from shifts and differences in the mutant load (Wallace et al., 2010). Indeed, due to stochastic segregation of mtDNA, the percentage of mutant and normal mtDNAs may drift during cellular divisions, and the percentage of the mutation load may vary drastically among the different tissues and organs, from 100% mutant load, defining homoplasmy, to the coexistence of mutant and wildtype copies, defining heteroplasmy. As the percentage of heteroplasmy increases, the energy production declines until the energy output falls below the minimum necessary for the physiological maintenance of cellular functions, causing the appearance of symptoms (Rossignol et al., 2003).

Until the development of next generation sequencing (NGS), molecular diagnosis of mitochondrial disorders was based on a combination of several techniques, including targeted Sanger sequencing for the detection of mutations, long-range polymerase chain reaction (PCR) and Southern blotting, for the detection of mtDNA rearrangements and depletions, whereas fluorescent PCR restriction fragment length polymorphism (RFLP) and pyrosequencing were used for the quantification of mtDNA variants and rearrangements (Moraes et al., 2003; Bannwarth et al., 2005; Wong and Boles, 2005). These techniques are still useful as confirmatory and independent tools to ascertain the presence of a given mtDNA variant identified by NGS. The development of massive parallel sequencing techniques now allows the systematic screening of the whole mitochondrial genome thus increasing the efficacy of the workflow with increased sample throughput and greater sensitivity in the detection of mtDNA variants (Vancampenhout et al., 2014; Ye et al., 2014; Seneca et al., 2015). However, massive parallel sequencing of the whole mitochondrial genome, with the increasing quantity and complexity of mtDNA data, led to difficulties to appreciate the variants identified, thereby posing new challenges in the molecular diagnosis of mitochondrial diseases. Indeed, several issues related to the interpretation of very low mutational loads (Guo et al., 2013), the discovery of variants of unknown significance (van der Walt et al., 2012), and mutations unrelated to the patient phenotype, generate difficulties in prioritizing the variants, and as a consequence different interpretations of mtDNA variants in the diagnostic process. This review provides an overview of the current strategies, databases and bioinformatics tools for an accurate annotation, prioritization and report of mtDNA variations coming from NGS, in order to carry out fast and accurate genetic counseling in patients with primary mitochondrial disease.

## mtDNA Variant Annotation

Careful mtDNA annotation of mtDNA variants is a prerequisite for accurate prioritization, addressed by several pipelines and online tools (Table 1A), like MSeqDR mvTool (Shen et al., 2018), Mitomaster (Lott et al., 2013), mtDNA-Server (Weissensteiner et al., 2016a), MitoTool (Fan and Yao, 2011), or SG-ADVISER (Rueda and Torkamani, 2017). Then, the general workflow for mtDNA variants prioritization could parallel that of the nuclear genome and rely on an accurate standardized annotation, based on consensus databases and *in silico* prediction tools.

**Table 1:** Online resources for annotation and prioritization of mtDNA variants.

**Tool**	**Website**		**References**
**A. ANNOTATION TOOLS AND PRIORITIZATION PIPELINES**			
MSeqDR MvTool	https://mseqdr.org/		Shen et al., 2018
Mitoseek	https://github.com/riverlee/MitoSeek		Guo et al., 2013
mtDNA-Server	https://mtdna-server.uibk.ac.at/index.html		Weissensteiner et al., 2016a
MITOMASTER	https://www.mitomap.org/foswiki/bin/view/MITOMASTER/WebHome		Lott et al., 2013
SG-Adviser	https://genomics.scripps.edu/mtdna/		Rueda and Torkamani, 2017
Mitotool	http://www.mitotool.org/		Fan and Yao, 2011
Mit-O-Matic	http://genome.igib.res.in/mitomatic/help.html		Vellarikkal et al., 2015
**Database**	**Specificity**	**Website**	**References**
**B. ONLINE DATABASES DEDICATED To mtDNA**			
HmtDB	mtDNA variants	https://www.hmtdb.uniba.it/	Clima et al., 2017
HmtVAR	mtDNA variants	https://www.hmtvar.uniba.it/	Preste et al., 2018
MITOMAP	mtDNA variants	https://www.mitomap.org/foswiki/bin/view/MITOMAP/WebHome	Kogelnik et al., 1996
Mitobreak	mtDNA rearrangements	http://mitobreak.portugene.com	Damas et al., 2014
EMPOP	Forensic database	https://empop.online/	Parson and Dur, 2007
*Mamit-tRNA*	tRNA variants	http://mamit-trna.u-strasbg.fr/	Putz et al., 2007
*PhyloTreemt*	Phylogenetic tree	http://www.phylotree.org/	van Oven and Kayser, 2009
**Database**	**Website**		**References**
**C. ONLINE GENERAL DATABASES INCLUDING mtDNA DATA**			
CLINVAR	https://www.ncbi.nlm.nih.gov/clinvar/		Landrum et al., 2016
CLINVAR Miner	https://clinvarminer.genetics.utah.edu/		Henrie et al., 2018
OMIM	https://www.omim.org/		Amberger et al., 2015
**Tool**	**Specificity**	**Website**	**References**
**D**. ***In silico*** **PREDICTION TOOLS**			
APOGEE	Coding variants	http://mitimpact.css-mendel.it	Castellana et al., 2017
MToolbox	Coding variants	https://github.com/mitoNGS/MToolBox	Calabrese et al., 2014
Mitimpact2	Coding variants	http://mitimpact.css-mendel.it/	Castellana et al., 2015
Mitoclass.1	Coding variants	https://github.com/tonomartin2/MITOCLASS.1	Martin-Navarro et al., 2017
MITOTIP	tRNA variants	https://www.mitomap.org/foswiki/bin/view/MITOMAP/MitoTipInfo	Sonney et al., 2017
PON-mt-tRNA	tRNA variants	http://structure.bmc.lu.se/PON-mt-tRNA/	Niroula and Vihinen, 2016
Haplogrep2	Haplogroup prediction	https://haplogrep.uibk.ac.at/	Weissensteiner et al., 2016b

### Population and Clinical Databases

Whereas mtDNA data are available from exome and genome sequencing data (Griffin et al., 2014; Patowary et al., 2017), the frequency of mtDNA variants in the general population is not reported in the databases such as GnomAD (Lek et al., 2016) or MARRVEL (Wang et al., 2017). The number of dedicated databases focusing on mtDNA with an active curation is limited (Table 1B), only three being available online: Mitomap (Kogelnik et al., 1996; Lott et al., 2013), HmtDB (Clima et al., 2017) and HmtVar (Preste et al., 2018). Together these resources gathered for instance more than 45,000 whole mtDNA sequences and over 70,000 mtDNA control region sequences for the Mitomap database. However, the interpretation of the variant frequency in the general population is difficult given that databases include patient data and because of the peculiarities of mtDNA genetics (incomplete penetrance, heteroplasmy level, influence of mitochondrial haplogroup background). For example, the m.3460G>A primary LHON mutation is reported 20 times in GenBank (Bentley et al., 2008) as of July 10, 2018 (Mitomap database), the pathogenic and unquestionable variant being reported both in patients, and also from phylogenetic studies. There is currently no consensus threshold to consider, often mtDNA variant is frequent in the population, thresholds between 0.2 and 0.5% being arbitrarily chosen in several studies (Wang et al., 2012; Lieber, 2013). However, they do not consider the variant frequency within a peculiar haplogroup which can lead to misinterpretation as some haplogroups are underrepresented in databases. For instance, Asian and African lineages represent only 21 and 13% of the Mitomap GB dataset. To overcome this problem, the Mitomap database warns if a variant is identified at >1% in at least one of the macro-lineages or over 10% in the major haplogroups for tRNA variants (Sonney et al., 2017). For the first step of prioritization, forensic databases such as EMPOP (Parson and Dur, 2007) are useful.

Clinical databases combining mtDNA variants and clinical files are available (Table 1C). General databases such as CLINVAR (Landrum et al., 2016), CLINVAR Miner (Henrie et al., 2018), or OMIM (Amberger et al., 2015) carrying information on mtDNA in addition to nDNA variants, can be distinguished from databases specifically dedicated to mtDNA such as Mitomap, HmtVar or HmtDB which are more exhaustive. In the latter, more than 10,000 variants are gathered throughout the whole mtDNA (Figure 1). For example in MT-ND1 gene only 42 variations are reported in CLINVAR, whereas 693 variants are available in Mitomap (Lott et al., 2013), and among them, 62 are reported associated with a clinical phenotype. MtDNA-specific information, such as heteroplasmy and haplogroup frequency are not systematically reported in each database, hence questioning the value of pipelines collecting information from multiple resources in order to provide a complete description of the variant. Other specialized databases are also helpful for variant prioritization: Mamit-tRNA database which compiles information on mammalian mitochondrial tRNA genes (Putz et al., 2007) or the Mitobreak database focusing on mtDNA rearrangements (Damas et al., 2014).

Even if the prevalence of mitochondrial diseases is in the order of 1/4,300 (Gorman et al., 2015), it was shown that at least 1 in 200 newborn cord bloods carry one of the ten most common pathogenic mtDNA mutations, which is much more frequent than expected (Elliott et al., 2008). Many of these mutations will probably be lost through stochastic segregation, but there is still a chance that these mutations will be transmitted to the offspring and causing mtDNA diseases in next generations. As mtDNA variations can be obtained from WES (Griffin et al., 2014) or WGS data, the identification of pathogenic variants unrelated to the patient's phenotype poses a challenge for the interpretation and report of the variant. Selected mtDNA confirmed variants, such as LHON (e.g., m.11778G>A, m.14484T>C, m.3460T>C or other rare LHON mutations as classified in the top 19 mutations, see mitomap) or deafness pathogenic variants such as the m.1555G>A, should be considered as secondary and actionable findings. As defined by the American College of Medical Genetics and Genomics which claimed that “the results of a deliberate search for pathogenic alterations in genes that are not apparently relevant to a diagnostic indication for which the sequencing test was ordered but which may nonetheless be of medical value or utility to the ordering physician and the patient” (Green et al., 2013; Kalia et al., 2017). As an example, we discovered in our clinical center the m.1555G>A variant in the *MT-RNR1*, responsible for the amino-glycoside-induced and non-syndromic hearing loss (Bitner-Glindzicz et al., 2009; Vandebona et al., 2009) in a family with isolated optic atrophy but without hearing impairment. The discovery of a pathogenic variant should be considered as “actionable,” i.e., pathogenic variants whose penetrance would result in medical recommendations and health care prevention. Indeed, the presence of the m.1555G>A may modify patient management, audiometric follow-up and hearing protection, prompting appropriate genetic counseling and recommendations with the avoidance of the use of specific drugs such as aminoglycosides (Estivill et al., 1998). Other mtDNA variants with unrelated or unclear relation to the clinical phenotype have been identified in the general population, such as primary LHON mutations found in patients, but unaccompanied by any ophthalmological sign (Inagaki et al., 2006; Yang et al., 2016). Incomplete penetrance is a hallmark of optic neuropathies, starting with LHON, and several parameters such as modifier genes or environmental factors such as tobacco smoking or the consumption of alcohol or administration of ethambutol, have been identified as risk factors for asymptomatic carriers of LHON mutations (Yu-Wai-Man et al., 2016; Caporali et al., 2017). Thus, some of the mtDNA pathogenic variants, such as LHON or deafness pathogenic variants should be carefully treated as “actionable mutations” and call for genetic counseling, especially to avoid exposure to risk factors and for a clinical follow up.

### *In silico* Prediction Tools and Pathogenicity Scores

The systematic mtDNA screening by NGS revealed a large number of novel variants of unknown significance (Lieber, 2013; McCormick et al., 2013), as exemplified by the identification of 11 patients among 71 from a pediatric cohort, harboring a novel variant of unknown significance (VUS) (van der Walt et al., 2012). In our diagnostic laboratory setting, about 6.5% of the patients carried a VUS. The interpretation of the clinical significance of mtDNA VUS is more complicated than for the nuclear VUS, in part due to the mtDNA characteristics, as heteroplasmy and high mutation rate (Marcelino and Thilly, 1999), which are not considered in the classical prioritization algorithms, and because of the limited guidelines for the mtDNA compared to those provided by the American College of Medical Genetics for nuclear VUS (Richards et al., 2015). Special efforts are currently underway with an initiative from an international group of mitochondrial researchers and clinicians to revise the ACMG guidelines specifically for mtDNA variants (Procaccio, personal communication).

*In silico* prediction tools, which evaluate the functional impact of variations using approaches based on interspecies sequence conservation and/or structure analysis, are currently the last step of variant prioritization. As prediction tools are specific for a type of variations, we will distinguish those dedicated to coding regions to those dedicated to other mtDNA regions.

**Figure 2:**
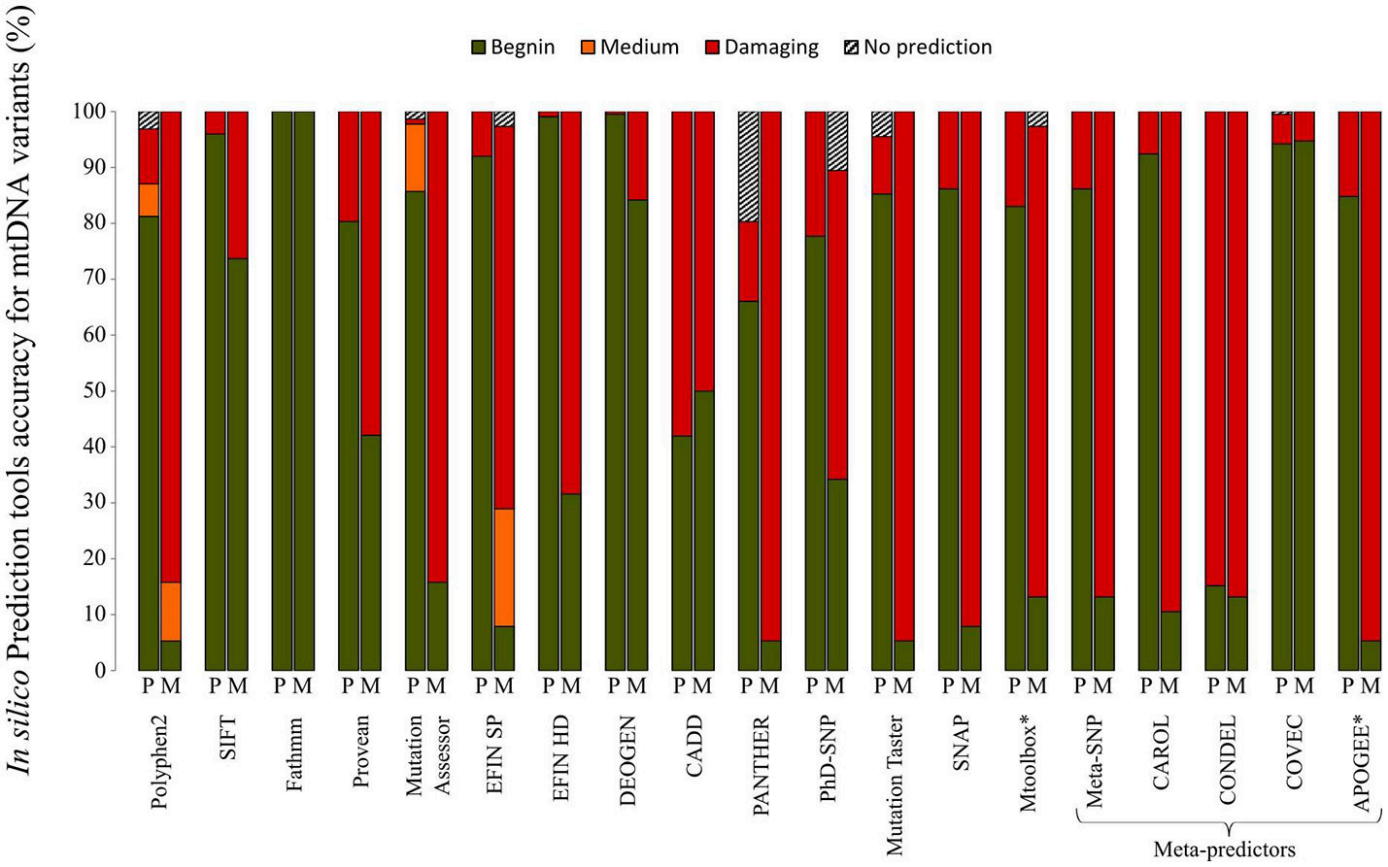
Performances of *in silico* prediction tools for non-synonymous mtDNA variants. A set of 38 confirmed pathogenic variants (M) and 224 non-synonymous variants classified as mtDNA polymorphisms (P) according to Mitomap, were assessed with 19 different prediction tools. Information about the different *in silico* tools is available at MitImpact2 website (http://mitimpact.css-mendel.it/). Variants were classified into 4 categories: benign (green), medium (orange), damaging (red) and no prediction (hatched) according to the tool prediction. Results are expressed as percentages. *In silico tools developed for mtDNA.

A plethora of *in silico* bioinformatics prediction tools exists for the prioritization of nuclear DNA coding variants. A thorough analysis of the main tools commonly used to evaluate the pathogenicity of variants demonstrated that the performances vary drastically when variants of the mtDNA-encoded proteins were tested. A set of 38 confirmed pathogenic variants (M) and 224 variants considered to be polymorphisms (P) according to Mitomap, were assessed with a set of 19 different prediction tools gathered in MitImpact2 (Castellana et al., 2015; Figure 2). Variants of the mitochondrial genome were subdivided into four categories based on the prediction of pathogenicity, i.e., benign, medium, damaging, and no prediction. The performances of the prediction of pathogenic mtDNA variants differed significantly between the different bioinformatics tools. For instance, more than 70% of the confirmed pathogenic mutations were predicted to be benign with SIFT, whereas about 15% of the pathogenic variants were not predicted as such by Polyphen2, highlighting that the tools developed for nDNA are barely suitable for mtDNA. Conversely, recent tools developed for mtDNA using machine learning based approaches (Table 1D) show better performances (Figure 2), as MToolBox (Calabrese et al., 2014), the meta-predictor APOGEE (Castellana et al., 2017), or Mitoclass.1 (Martin-Navarro et al., 2017), confirming the need to pursue the development of tools dedicated to mitochondrial genetics.

Few tools are dedicated to mitochondrial tRNAs, accounting for nearly 50% of the mtDNA alterations identified in patients (Schaefer et al., 2008; Gorman et al., 2015). PON-mt-RNA is a multifactorial score associating 12 features including evolutionary conservation, primary to tertiary structures, and functional assays including biochemistry and histochemistry (Niroula and Vihinen, 2016). All the precomputed data are downloadable at http://structure.bmc.lu.se/PON-mt-tRNA/datasets.html/. MITOTIP, the most recent tool available through Mitomap combines conservation data, structural analogies with other tRNA variants and secondary structure information (Sonney et al., 2017), giving the best prediction performances in terms of sensitivity and specificity. However, specific tools for the interpretation of VUS in *MT-RNR1* and *MT-RNR2* combining conservational information with functional and structural data are helpful to better interpretation of ribosomal variants (Smith et al., 2014; Elson et al., 2015).

Thus, many bioinformatics tools are useful for predicting the functional impact of mtDNA variants, nevertheless results should still be considered with caution, due to the high rate of false negative and false positive predictions as demonstrated in Figure 2. To overcome this problem and improve the prioritization of mtDNA VUS, several teams have developed scoring approaches (McFarland et al., 2004; Mitchell et al., 2006; Wong, 2007). These scores which combined algorithms similar to those of *in silico* prediction tools (i.e., structure, conservation) and functional *in vivo* and *in vitro* evaluation show better performances, but their use is still limited, because functional studies are time-consuming and tissues such as fibroblasts, cybrids, or muscle samples, are not always available for assessing the consequences of the variants on mitochondrial physiology. Given the increasing number of variants of unknown significance identified by NGS, it would be interesting to regularly re-evaluate these pathogenicity scores based on new information.

### Heteroplasmy Level Interpretation

The development of NGS techniques and dedicated bioinformatics pipelines (Calabrese et al., 2014; Weissensteiner et al., 2016a; Marquis et al., 2017) has widely improved the detection of low-level mtDNA variations. While the major drawback of Sanger sequencing is its lack of sensitivity for detecting DNA mutant loads lower than 20% (Procaccio et al., 2006; Wong, 2010), the limit of detection (LOD) of NGS strategies is considerably lower for the major NGS technologies with an LOD close to 5% for pyrosequencing methods (Zaragoza et al., 2010; Sosa et al., 2012) and semiconductor technology (Huang, 2011; McElhoe et al., 2014; Vancampenhout et al., 2014; Seneca et al., 2015), and close to 1% for reversible terminated chemistry (Huang, 2011; Zhang et al., 2012). Recently, the development of duplex sequencing further improved the power to detect low-level of heteroplasmy down to 0.01% (Schmitt et al., 2012; Ahn et al., 2015).

One of the first pitfalls to this gain of sensitivity is the difficulty to confirm very low heteroplasmy mtDNA variations, eliminating possible sequencing artifacts, which could impair the diagnosis accuracy and prevent sound genetic counseling. Several sensitive technics have been developed such as fluorescent PCR-RFLP (Procaccio et al., 2006; Bannwarth et al., 2013), PNA clamp PCR (Urata et al., 2004), digital or real-time PCR (He et al., 2002; Grady et al., 2014). Thus nowadays, although no consensus threshold has yet been defined, previous studies establishing the detection limit from 1 to 10% according to the technology used (Cui et al., 2013; Wong, 2013; Seneca et al., 2015). The detection of low mutation loads improves the diagnosis of mitochondrial diseases and the quality of genetic counseling, particularly for mutation carriers. However, the clinical relevance is sometimes difficult to interpret in probands, with the risk of false conclusion of the implication of mtDNA in the disease, instead of a nuclear gene variant. For example, a report described a patient presenting the Alper's syndrome, carrying both nuclear and mtDNA mutations: two pathogenic variants in *POLG* and also the m.3243A>G at 8% mutation load in blood (Tang et al., 2013). The mtDNA mutation was probably not responsible for the phenotype, considered as secondary finding, but may potentially modulate the clinical phenotype as described (Tang et al., 2013). Indeed, it is commonly accepted that mtDNA mutations have clinical consequences only over a certain heteroplasmy level, also called threshold effect (Rossignol et al., 2003). It was recently shown that clinical phenotypes are associated with low heteroplasmic mtDNA pathogenic variants (Ng et al., 2018) or deletions (Leung et al., 2018), and additional low-level heteroplasmic variants can explain the phenotypic variability of mtDNA homoplasmic mutations (Ballana et al., 2008). Unfortunately, mtDNA databases do not specify the level of heteroplasmy leading to clinical phenotype in patient, with the exception of Mitomap (Kogelnik et al., 1996; Lott et al., 2013), which provides partial information mentioning the homoplasmic or heteroplasmic nature of pathogenic variants. So, there are different situations to consider depending on the mtDNA variation and the tissue analyzed in the proband. When the mutation has been identified from a blood sample, or from the analysis of more relevant tissues, such as muscle or uroepithelial cells (McDonnell et al., 2004; Blackwood et al., 2010; Liu et al., 2013; Fayssoil et al., 2017; Grady et al., 2018), the presence of the heteroplasmic mutation can be linked to the phenotype of the patient. Indeed, due to the stochastic segregation of mtDNA, mutation loads can drastically vary in-between and within tissues, and several mutations may undergo selection in blood cells, as for example the m.3243A>G, for which heteroplasmy decreases by 1.4% per year in blood (Rahman et al., 2001). Recently, a new algorithm was developed to estimate the m.3243A>G mutation heteroplasmy in muscle based on the quantification in blood or uroepithelial cells (Grady et al., 2018). This tool available through an online webserver (https://newcastle-mito-apps.shinyapps.io/m3243ag_heteroplasmy_tool/) is then helpful for the clinicians for the interpretation of low-level of the m.3243A>G mutation identified in peripheral tissues. Coupling the mtDNA copy number with mutant load quantification is another argument to assess the clinical variability (Frey et al., 2017; Emperador et al., 2018). Indeed, an increase of the mtDNA copy number in a heteroplasmic situation will modify the absolute value of the wild type mtDNA copies, even if the mutant load remains unchanged and therefore may explain the variability of the clinical phenotypes of mtDNA-related disorders, as shown for LHON (Giordano et al., 2014) or MELAS syndrome (Liu et al., 2013; Grady et al., 2018). The gain of sensitivity enabled by massive parallel sequencing also allows identifying high level of heteroplasmy, in samples that were initially considered as homoplasmic (Genasetti et al., 2007; Ballana et al., 2008; Carrasco Salas et al., 2016). The detection of heteroplasmy has always been considered as strong argument for the variant scoring pathogenicity (McFarland et al., 2004; Mitchell et al., 2006).

## Perspectives: Toward an Integrative Analysis of the Mitochondrial Genome

With the development of NGS, we have now access to the entire mtDNA sequencing information. Therefore, additional information such as mitochondrial haplogroups, identification of helper or synergistic mutations and co-occurrences of variants should be incorporated in clinical diagnostic settings, as they are thought to modulate the phenotypic expression of mtDNA pathogenic variants.

### Influence of Mitochondrial Haplogroups

Mitochondrial haplogroups, i.e., clusters of nucleotide polymorphisms accumulated in mtDNA during human evolution and transmitted through maternal lineage, play a role in modulating the penetrance of mitochondrial diseases (Ghelli et al., 2009; Gomez-Duran et al., 2012), or in age-related disorders (van der Walt et al., 2004; Wolf et al., 2010; Hudson et al., 2013). Haplogroups are defined by ancient sequence polymorphisms that occur at the base of a particular branch of the mtDNA phylogenetic tree (Ingman et al., 2000). For example, the higher prevalence of specific subclades of haplogroup J have been shown to modify the pathogenicity and penetrance of LHON (Brown et al., 2002; Ghelli et al., 2009; Caporali et al., 2017). Computing mitochondrial haplogroups from NGS data is relatively easy, as many bioinformatics tools (Tables 1C,D) have been developed based on the PhyloTree data (van Oven and Kayser, 2009) such as HaploGrep2 (Weissensteiner et al., 2016b), Mitomaster (Lott et al., 2013), or HmtDB (Clima et al., 2017) available on a web-server, or integrated into an all-in-one pipeline as in MToolBox bioinformatics suite (Calabrese et al., 2014), MseqDR mvTool (Shen et al., 2018), mit-o-matic (Vellarikkal et al., 2015). Conversely, the Phy-Mer software allows the classification of haplogroups from the FASTQ files, i.e., without prior alignment, avoiding mistakes caused by artifactual sequencing variants (Navarro-Gomez et al., 2015). However, few databases provide information about the prevalence of a variant in a specific haplogroup. Mitomaster allows a quick overview of the variant distribution in the different haplogroups, while HmtDB through multiple queries gives the frequency of a pathogenic variant within the haplogroup.

### Co-occurrence of mtDNA Variants

Even with well-characterized mitochondrial phenotypes such as LHON or MELAS, shown to be associated with confirmed mtDNA variants, such as the m.11778G>A or m.3243A>G, respectively, mitochondrial whole genome screening may provide additional information with the co-occurrences of mtDNA variants that may modulate the phenotype (El Meziane et al., 1998; Khan et al., 2013). For example, the presence of the heteroplasmic m.12300G>A variant in *MT-TL2*, with a mutant load of about 10%, was shown to suppress the mitochondrial dysfunction in transmitochondrial cybrid cells carrying the m.3243A>G mutation with 99% mutated mtDNA, emphasizing the need for a complete mtDNA screening (El Meziane et al., 1998). A large study analyzing the distribution of known disease-causing mutations in a set of more than 30,000 mtDNA sequences has recently suggested that the mtDNA background influences the development of mtDNA mutagenesis with the acquisition of recurrent mtDNA variants (Wei et al., 2017). In addition, it was recently shown that, apart from any pathogenic mtDNA variants, the combination of rare non-synonymous polymorphisms could lead to LHON, as exemplified by both combinations of variants m.14258G>A in the *MT-ND6* gene (p.Pro139Leu) and m.14582A> G (p.Val31Ala); m.14258G> A, m.10680G> A in the *MT-ND4L* gene and m.12033A> G in the *MT-ND4* gene (Caporali et al., 2018). Functional studies of cybrid cells carrying both variant combinations revealed that the biochemical deficiency was transferred to mutant cybrids. Unfortunately, currently databases and bioinformatic pipelines do not allow identifying rare co-occurrences of variants, and further developments of these databases are needed to implement a searchable function of possible combinations of mtDNA variants.

### Influence of the Nuclear Genome

As mitochondria are driven by two genomes, several studies have demonstrated that nuclear variants may modulate the phenotypic expression of mtDNA pathogenic variants (Davidson et al., 2009; Jiang et al., 2016). For example, it has recently been suggested that the c.572G> T variant (p.Gly191Val) in *YARS2*, a gene coding for mitochondrial tyrosyl-tRNA synthetase was associated with a mitochondrial protein translation defect, worsening mitochondrial respiratory chain deficiency in patients carrying the m.11778G>A LHON mutation (Jiang et al., 2016). Thus, *YARS2* appeared as a nuclear modifier, capable of triggering optic atrophy in individuals carrying the m.11778G>A mutation, and would explain the incomplete penetrance of LHON, in addition to other parameters or environmental factors (Dimitriadis et al., 2014; Giordano et al., 2015). Unfortunately, major databases such as Mitomap, HmtDB, and HmtVar do not currently allow the search of the co-occurrence of mtDNA variants or in combination with nuclear variants. As the mtDNA data can be extracted from exome or genome sequencing data (Griffin et al., 2014), these information could be integrated in general databases such as GnomAD.

The integration of additional information in mitochondrial databases or in the filtering and prioritization process of bioinformatics pipelines, such as haplogroups, co-occurrences of mtDNA or nuclear variants shown to modulate the phenotype should be very helpful to assess the pathogenicity of a given variant for a better interpretation and as a possible explanation for incomplete penetrance or phenotypic variability. Special efforts should be directed at developing bioinformatics tools dedicated to the mitochondrial genome such as MseqDR.

## Conclusion

Due to NGS technologies the amount of mtDNA is now constantly increasing and special efforts from the mitochondrial and scientific community have to be made to collect and organize the large quantity of generated information. In addition, the complexity of mtDNA interpretation is increasing in an exponential manner, requiring better and specific prediction tools to assess mtDNA variant pathogenicity or to assess the co-occurrence of variants.

## Author Contributions

CB and VP decided on the content and structure of the first version of the manuscript. CB, DG, VD-D, EC, PA-B, and MC collected the information. VP and CB drafted the first and final version of the manuscript while DG, GL, DB, and PR revised the manuscript.

### Conflict of Interest Statement

The authors declare that the research was conducted in the absence of any commercial or financial relationships that could be construed as a potential conflict of interest.
